# Can a simple chemical help to both prevent and treat sepsis

**DOI:** 10.1186/s13054-018-2161-3

**Published:** 2018-09-29

**Authors:** Anitra C. Carr

**Affiliations:** Department of Pathology and Biomedical Science, University of Otago, Christchurch, New Zealand

I was pleased to read the commentary by Kempker et al., ‘Sepsis is a preventable public health problem’ (*Crit Care*, 2018, 22:116). Prevention of a critical illness such as sepsis is always preferable, for both the individual patients and the health system in general, with the cost of treating sepsis and its long-term health disabilities contributing a significant personal and societal burden. It has long been known that individuals who are vitamin C deficient are more prone to severe illnesses such as acute respiratory infections, with pneumonia being a major cause of death for individuals with the deficiency disease scurvy [1]. Acute illnesses, such as sepsis, can also impact negatively on vitamin C status, despite recommended enteral and parenteral intakes [2]. Due to the increased demand and utilization of vitamin C during critical illness, administration of at least 2–3 g/day of vitamin C is required to replete the plasma of these patients. Thus, inadequate vitamin C status may be both a contributor to and a consequence of severe illness.

**Fig. 1:**
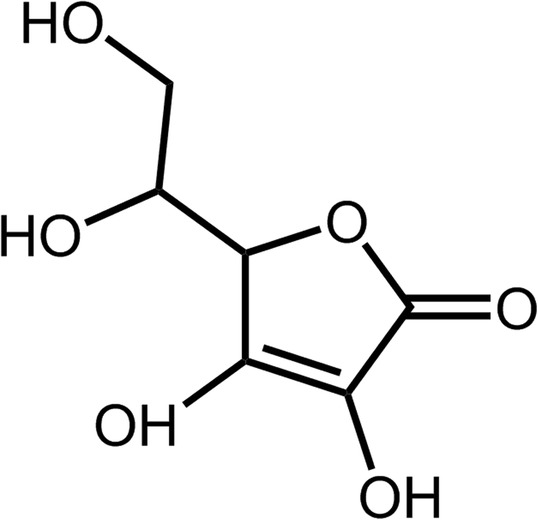
Chemical structure of vitamin C (ascorbic acid), a molecule essential to life due to its numerous biosynthetic and regulatory roles in the body

**Fig. 2:**
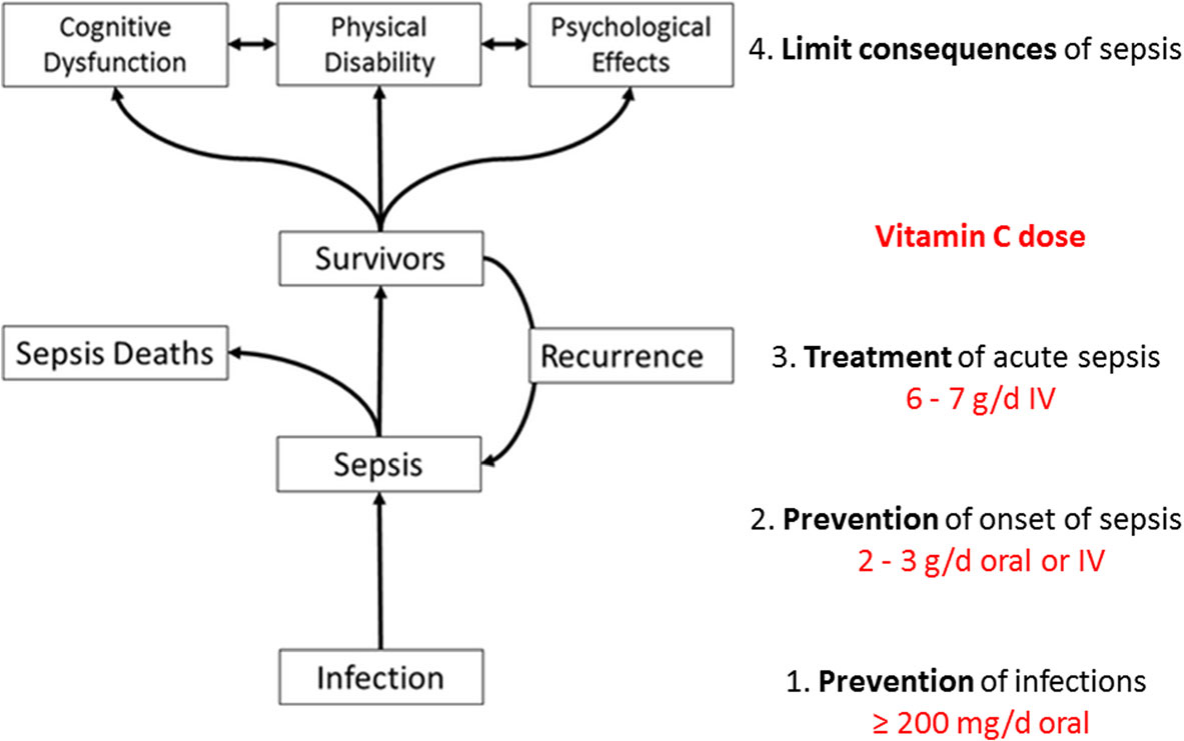
Prevention and treatment of infections and sepsis by vitamin C. Figure adapted from Kempker et al., *Crit Care*, 2018, 22:116. *IV* intravenous

Vitamin C is a small, simple carbohydrate-like compound with a molecular structure that facilitates donation of electrons resulting in its ability to act as a potent antioxidant and enzyme cofactor with pleiotropic biosynthetic and regulatory functions in the body (Fig. 1) [3]. Meta-analysis has indicated that prophylactic intake of vitamin C (0.2–2 g/day) can reduce the incidence of common respiratory infections by up to 50% in individuals who are exposed to enhanced physical stress, and can also decrease the incidence of more severe respiratory infections, such as pneumonia, a major cause of sepsis (Fig. 2) [4, 5]. A number of small clinical studies carried out over the past few years have also indicated that administration of low gram doses of vitamin C can improve the clinical outcomes of patients with sepsis and septic shock, including potentially decreasing mortality (Fig. 2) [1]. Although the long-term outcomes of these patients have not been assessed, continued supplementation with vitamin C would likely also attenuate long-term disability.

Vitamin C is very cheap and remarkably safe, being a water soluble vitamin which is readily excreted by the kidneys or removed by haemodialysis. Although more studies clearly need to be carried out, the preliminary findings indicating both prevention and treatment of severe infections and sepsis by vitamin C are encouraging.
